# Older patients’ experiences of access to and use of e-consultations with the general practitioner in Norway: an interview study

**DOI:** 10.1080/02813432.2022.2161307

**Published:** 2023-01-02

**Authors:** Eli Kristiansen, Helen Atherton, Bjarne Austad, Trine Bergmo, Børge Lønnebakke Norberg, Paolo Zanaboni

**Affiliations:** a Norwegian Centre for E-health Research, University Hospital of North Norway, Tromsø, Norway; b Department of Clinical Medicine, UiT The Arctic University of Norway, Tromsø, Norway; c Unit of Academic Primary Care, Warwick Medical School, UK; d General Practice Research Unit, Department of Public Health and Nursing, Norwegian University of Science and Technology, Trondheim, Norway

**Keywords:** E-health, e-consultation, general practice, qualitative research, older patients, access

## Abstract

**Objective:**

To explore older patients’ experiences with accessing and using e-consultations to send text-based clinical inquiries to the general practitioner (GP) online.

**Design:**

Qualitative study based on semi-structured interviews. Results were analysed through a six-phase thematic analysis and interpreted through Levesque’s framework of patient-centred access to health care.

**Setting:**

General practice in Norway.

**Subjects:**

Patients aged over 65 years (*n* = 16) with experience in using e-consultations.

**Results:**

Respondents considered e-consultations as an integrated part of general practice which helped them achieve better access to health care. We identified four themes describing older patients’ access to and use of e-consultations: 1) the importance of digital health literacy to learn about and use the service – and the fear of losing it, 2) the high availability of the service as the main advantage, due to the perceived unavailability of physical GP services, 3) the importance of voluntary use of e-consultations, 4) the importance of a trusting relationship with the GP.

**Implications:**

Information about e-consultations and guidelines for suitable use are recommended to ensure equal access to all patients, regardless of their digital health literacy. Availability problems and high work burdens for the GPs could affect the patients’ choice for using e-consultations. If e-consultations are used for triage purposes, caution should be taken to avoid a shift in workload from the health secretary to the GP.

**Key points of article**
- The extended use of e-consultations with the general practitioner has raised concerns that the service may not be accessible and suitable for older patients.
- For older users, e-consultations can represent a positive addition to physical consultation forms due to the high availability of the service in a general practice setting characterised by long waiting times.
- Digital health literacy is essential to learn about and use the service. Information about the service and how to use it should be available to all patients to ensure equal access.
- A trusting relationship with the GP is described as essential for older patients to perceive the outcome of e-consultations as appropriate and safe.

## Introduction

E-consultations with the general practitioner (GP) have been shown to produce several benefits to patients, including eliminating travel, reducing the spread of infectious diseases and saving time [1,2]. However, the extended use of e-consultations in recent years has created a debate about how digital services can affect the principle of equality in health care as access to e-consultations can be harder to achieve for some patient groups [3–5]. In particular, older patients often have a higher need for health care and a higher risk of complex health conditions and multimorbidity. At the same time, they tend to have a lower digital health literacy compared to the general population [6]. Digital health literacy is defined as the capabilities and resources required for individuals to use and benefit from digital health resources [7]. Consequently, e-consultations can be less accessible and suitable for older patients [8,9].

E-consultations are an asynchronous service used by patients to send a text-based clinical inquiry to their GP through an online patient portal. In Norway, offering e-consultations is recommended by the health authorities, but not mandatory. There are no official statistics regarding how many GPs currently offer e-consultations. The use of e-consultations in Norway increased significantly after the COVID-19 pandemic emerged in March 2020. In December 2021, e-consultations, video consultations and telephone consultations^1^ together accounted for 22.7% of all consultations with the GP for patients aged over 60 years. This was noticeably lower than the national average of 33.8% [10].

Other countries have implemented e-consultations in ways that differ somewhat from Norway. In Denmark, the adoption of e-consultation is higher than in Norway, particularly amongst older patients [11], and the service has been mandatory since 2009. Studies have demonstrated that Danish patients use the service mainly for non-complex issues, but also for sensitive issues if the relationship between doctor and patient is established and based on trust [12]. Danish patients are overall satisfied with the flexibility of the service [11,12]. In England, GP practices are also required to offer and promote non-physical consultations for their patients [13]. Online consultations in England differ from e-consultations in Norway, as they are typically used to direct patients to the appropriate level of care. Nonetheless, the service has not necessarily been shown to give patients better access to health care [14]. Little research has been conducted on how e-consultations are used in the Norwegian context and how the accessibility of the service is perceived by patients.

GPs have expressed concern that e-consultations might be too easily available for relatively healthy individuals [3,15], with a consequent risk of skewing the health care offer towards patient groups who do not necessarily need it the most. A fundamental value in the Norwegian health care system is to minimise inequities in access to health care, with one of the core values of the Nordic General Practice stating that patients with the greatest need for health care should be prioritised [16]. According to Levesque’s framework of access, access to a health care service is not merely measured by the supply of the service but by the extent to which a patient population ‘gains access’ and uses the service [17]. To be able to use the service supplied, patients must possess essential abilities. These patients’ abilities, in combination with characteristics of the supply, describe the accessibility of the health care service. Yet, there is limited evidence regarding the consequences of offering e-consultations in relation to equity [18] and access to health care by older patients [19]. Studies investigating patients’ perception and use of e-consultations have shown that patients appreciate the high availability [11,20,21] but also point out that the service is mainly used by younger patients and may not suit older patient groups [15,20]. By investigating older users’ perspectives of the service, we can translate their experiences into valuable understanding of the accessibility in this patient group. This knowledge, in turn, is valuable for improving access to the service among older patients.

Aim: This study aimed to explore older patients’ experiences with accessing and using e-consultations to send text-based clinical inquiries to the GP online.

## Design, materials, and methods

### Study design

The qualitative design of the study allowed for an inductive approach, which is appropriate for exploring the individual experiences of the phenomenon. We conducted semi-structured interviews to obtain rich descriptions of how older patients access and use e-consultations with their GP.

### Setting

In Norway, the GPs are organized in a GP scheme, giving all inhabitants the right to have a GP. E-consultations with the GP are delivered either through the national health portal helsenorge.no, or through private digital platforms. If offered, the service is typically always available online, day and night. By law, GPs should normally answer the patient within five days [22]. Patients are charged the same out-of-pocket fee for an e-consultation as for a physical consultation. Patients who pay more than a certain amount in user fees for public health services in a year (NOK 2921 in 2022) [23] receive an exemption card and do not have to pay user fees, including those for e-consultations, for the rest of the year.

### Data collection

Criteria for participation in the study were being aged 65 or older and having experience with using e-consultations. We did not set any exclusion criteria for participation, as we wanted to gather experiences from a diverse group of older users. While we aimed to obtain variation in gender and old age, it turned out to be harder to recruit men than women. Participants were recruited through different recruitment strategies: advertisement in magazines for members of the Pensioners’ Association (*n* = 1), written handouts about the project and presentations at meetings of different organisations for pensioners (e.g. the Pensioners’ Association, Senior Net and the Red Cross Senior group) (*n* = 0), and a targeted advertisement on Facebook (*n* = 12). Because users of e-consultations have a certain level of digital health literacy and, as such, are likely to be active on social media, the use of social media for recruiting participants was deemed appropriate to the scope of this study. Due to the lack of men recruited by the aforementioned recruitment strategies, we asked one GP office to help us recruit men with experience in using e-consultations (*n* = 3).

An inductive, semi-structured interview guide with open-ended questions about health care needs, level of digital health literacy and use of e-consultations was developed by the author group to explore how users achieved access to the service (supplementary file 1). All interviews were conducted by phone by the first author (EK) in November and December 2021 and lasted between 30 and 60 min. As the national authorities were still urging social distancing due to the COVID-19 pandemic during the period of the interviews, we chose to conduct the interviews by phone for safety reasons. This also made it easier to reach participants from the entire country. The interviews were transcribed verbatim. We interviewed until we reached sufficient information power (i.e. no essential further information about the use of and access to e-consultations was found) [24].

### Data analysis and interpretation of results

The analysis was performed by an interdisciplinary research team with backgrounds in general practice, health service research, technology, and health economics. All authors had knowledge about and interest in e-health. Before the study, there was an outspoken perception that e-consultations may not be particularly suited for older patients who often have complex health issues and low digital health literacy. The analytic discussions were placed in the balance between knowledge from general practice (BA and BLN) and e-health research (EK, HA, TB, PZ). All authors had experience with qualitative analysis. We used an inductive data-driven six-phase thematic analysis [25] as suggested by Braun and Clarke [26]. All authors with Norwegian as the main language (EK, BA, TB, BLN, PZ) read all the interviews to become familiar with the data and presented their preliminary suggestions for codes. These were discussed and all interviews were then coded. The main author (EK) coded all interviews, while co-authors were assigned selected interviews so that all the interviews were coded by two persons. The authors developed and modified the preliminary codes throughout the process and new codes and inconsistencies in coding were discussed in the group. The whole group agreed upon the final coding. We then searched for relevant themes within the coding. The whole research team participated in suggesting and discussing themes, and finally, we agreed upon four main themes [see Table 1]. EK summarized the results and all co-authors suggested analytic improvements. The final revision was done by EK and PZ. Transcripts were analysed using NVivo (version 12).

**Table 1.** Overview of final coding and development to main themes.

Names of final codes:	Developed into the following main themes:
Adoption of service	The importance of digital health literacy and the fear of losing it
Patient autonomy	The importance of digital health literacy and the fear of losing it
Digital health literacy	The importance of digital health literacy and the fear of losing it
Concern for other old people and own future	The importance of digital health literacy and the fear of losing it
Self-triage	Importance of voluntary use of e-consultations
Suitability of other consultation forms	Importance of voluntary use of e-consultations
Suitability of e-consultations	Importance of voluntary use of e-consultations
Non-suitable situations for e-consultations	Importance of voluntary use of e-consultations
Availability of the service	High availability of service as the main advantage
Patient-provider relationship	The importance of a trusting relationship with the GP
Communication through e-consultation	The importance of a trusting relationship with the GP

After conducting the empirical analysis, the findings of the study were interpreted using Levesque’s framework of patient-centred access to health care [17]. This framework sees access to health care as a process that depends on five dimensions of accessibility regarding the suppliers (approachability, acceptability, availability, affordability and appropriateness) and five corresponding abilities (ability to perceive, seek, reach, obtain and engage) of patients necessary to gain access to and use the service. Based on this framework, we identified important requirements necessary to provide e-consultations as an equally accessible service for all patients.

## Results

There were 13 women and 3 men among the 16 respondents. The respondents had different health care needs, but all, except one, had higher health care needs (e.g. number of annual contacts with the GP) than the average Norwegian citizen [see Table 2] [27].^2^ Several of the respondents had used e-consultations for many years, and the majority had used the service since before the COVID-19 pandemic.

**Table 2.** Characteristics of respondents (*n* = 16).

Characteristics of respondents		n
Age (years)	65–69	6
	70–75	5
	76–80	3
	over 80	2
Time in retirement (years)	Still working	3
	1–3 years	4
	4–8 years	3
	More than 8 years	6
Place of residence	Urban > 70,000 inhabitants	9
	Rural < 70,000 inhabitants	7
Number of contacts with GP (last year)	1–2 3–5 More than 5	1 5 10

We identified four themes describing older patients’ use of and access to e-consultations: 1) the importance of digital health literacy and the fear of losing it, 2) the high availability of the service as the main advantage, 3) the importance of voluntary use of e-consultations, 4) the importance of a trusting relationship with the GP.

### The importance of digital health literacy and the fear of losing it

The respondents had a relatively high level of health literacy and a desire to keep track of and manage their own health. They also possessed confidence in their own abilities to follow up on their health and spent a fair amount of time doing so. Several respondents described that they prepared themselves and searched for information before appointments with their GP. They explained that using digital resources helped them to follow up on their health and they perceived e-consultations as a tool to achieve needed health care.

My regular medication is only valid for a year, they are prescribed one year at a time. It is a lot to take care of all the time, so I spend a lot of time online. (#7; female 68 years old)

The respondents highlighted the importance of having enough digital health literacy to learn about the service and be able to use e-consultations. None of the respondents had received information about or been encouraged to use e-consultations by their GP; they had all adopted the service unsolicited. Their level of digital health literacy gave them the confidence to navigate the online health portal and try new digital services.

E-consultations were rarely the first digital service the users adapted to communicate with the GP office. Many of the respondents were already acquainted with electronic prescription renewal and, whilst using that service, they encountered the possibility of sending e-consultations to the GP. Others became aware of e-consultations after receiving a digital message from the GP (e.g. regarding test results).

No one in the doctor’s office said anything to me [about the service], so it was probably when I looked around at ‘helsenorge.no’ [the official Norwegian health portal] that I learned about the possible to send a message to the doctor. I was curious to see how it worked, so I sent a small message, and there it was, an answer the day after. (#16; female 77 years old)

Using digital services to stay or get in contact with the health care system was described as positive and, for most, a choice they had made for themselves. However, some respondents felt pressured to use technology to maintain contact with the health care system. ‘… I’m afraid of being left behind’ said one respondent (#11; female 69 years old) when explaining why she started using e-consultations to communicate with her doctor. Some respondents were concerned about other old people who lacked digital health literacy. In addition, they worried about a possible decline in their own digital literacy in the future. The possible manifestation of health issues which often come with old age (e.g. low vision, hearing loss, trembling hands or cognitive impairment) were feared as potentially affecting their ability to use and keep up with digital health services.

I want to continue using it [e-consultations] as long as I am capable to. I can see that there will be challenges if I get dementia. Or if you get low vision, for example. I can see that there can be challenges with age and disability of the senses. (#10; female 69 years old)

### High availability of the service as the main advantage

As the e-consultation service was always available online, without waiting time or hourly limitations, the respondents felt that the service provided increased availability of health care services. This was considered the main advantage of the service. The users perceived the GP as overloaded with work and experienced long waiting times at the GP office, especially over the phone. Even the respondents with low mobility did not express that getting to the GP office for consultation was particularly stressful, however, the long waiting time on the phone to contact the GP office was perceived as unsatisfactory. E-consultations were considered a much more convenient option. The respondents explained that it was easier to contact the GP through e-consultations compared to physical consultations because the service did not depend on the GP’s availability.

If I got something urgent in the evening and wondered if I should goin [to the GP's office] the next morning, for example. In these situations, there can often be a long waiting time on the phone, and then it is much easier to write a text online. I get the impression that they check the internet often. (#10; female 69 years old)

The respondents expected a short response time on e-consultations. Based on their experience, they said that inquiries were normally answered within one or two days. One respondent said that, whilst she knew that, by law, an e-consultation must be replied to within five days, she would be annoyed if she had to wait more than a day for an answer. Another respondent contacted the GP with acute questions, as he felt sure he would receive an answer within the next day. The high availability of the service, together with the absence of waiting time to send an inquiry and the short response time for an answer, was described as the main reasons for the high satisfaction with the service.

… if I have an acute issue and send a text, then I know I would get an answer the next day with an instruction on doing either this or that. (#15; male 69 years old)

### The importance of voluntary use of e-consultations

E-consultations played an important role in giving patients the possibility of being actively involved in managing their health and it were perceived as useful when the patients felt in control of deciding when to use it. Whilst e-consultations were appreciated, it was pointed out that they could not replace all physical appointments. The respondents emphasised that the use of e-consultations should be voluntary for patients, and no limitations for booking appointments with the GP in person should be introduced. The respondents argued that everyone should be allowed to see the doctor in person if they wanted to, and no one should be forced to do an e-consultation, even if the doctor thought it was enough to clarify the problem. They believed there were fewer misunderstandings and less chance that the doctor could miss something important by meeting in person. Consequently, physical consultations were considered safer than e-consultations for severe issues. The respondents expressed concerns that GPs could prefer e-consultations if they perceive them as a more efficient consultation form, thus making physical consultations less available. E-consultations were described as a support for self-help and patient empowerment if the criteria of voluntary use without restrictions for physical consultations was fulfilled.

He [the GP] contacted me in writing, but I thought I should meet him. I thought it would be good to talk to him about certain things and we could discuss what he thinks and means, and eventually we could agree. (#11, woman 67 years old)

The respondents were divided when reflecting on whether e-consultations replaced office visits or were used in addition to physical consultations. The word ‘extra service’ was used, and the threshold seemed lower for e-consultations than physical consultations. Using e-consultations to clarify whether a doctor’s appointment was needed right away was common. However, some respondents said that they sent e-consultations with content that was considered so important they would have booked a physical appointment if they had not had the opportunity for e-consultation. Others explained that they used e-consultations for following up on test results, medication use and less severe health issues.

I would say an e-consultation is instead of a physical appointment. I feel like I should not take up the doctor’s time by coming to the office, and it is just so much easier to write a text. If it is not something very special, I can just as well take it online. (#8; female 82 years old)

The respondents believed in their ability to assess which health issues were suitable for e-consultations. However, on some occasions, this was perceived to put too much responsibility on the patient. For this purpose, some believed that GPs, based on their clinical knowledge, should be able to decide whether a consultation should be done digitally or physically. None of the respondents was aware of specific instructions on how to use e-consultations, and they suggested that providing technical guidance and/or guidelines to older people could help them achieve access and facilitate suitable use.

E-consultations have probably come to stay and there will probably be more digital possibilities, so maybe we should get some written information about it, so there are clear rules. (#9; female 75 years)

### The importance of a trusting relationship with the GP

Most of the respondents described a trusting relationship with their GP. Both having met their GP several times and their GP having prior knowledge about their illnesses and history were described as factors creating a trusting relationship. The respondents believed that when the patient and GP had an established relationship, it was easier for the patient to describe a health problem in a text, and for the GP to capture the situation and the importance of what was described. Furthermore, the patients seemed to have less concerns of misunderstandings and more trust in the outcome of the e-consultation, if it was sent to a GP they knew from before.

I feel reassured [about e-consultations] because she [the GP] knows me so well. But then again, I have tremendous trust in the doctor’s office and the doctor, so maybe because of that, I trust what she says, without having to sit physically in the office with her. (#15; male 69 years old)

A trusting relationship with the GP could also affect the overall use of e-consultations, as some would not send e-consultations if their regular GP was away, even if the substitute doctor or another GP in the practice could answer them. This was explained by a lack of trust in others than their regular GP when using e-consultations. It was also described that, if patients had to change GP, they would want to get to know the new GP through physical consultations to establish a trusting relationship before using e-consultations. The patients’ awareness of the GP’s high workload could affect their use of e-consultations. The respondents described a self-imposed sense of responsibility to choose e-consultations, as they were perceived as less time-consuming for the GP.

I know he’s terribly busy. Cruelly busy. Therefore, I write it down and send questions to him, so he can look at it when it fits him. (#1; female 96 years old)

At the same time, the respondents described physical consultations with stressed GPs, where they felt as if they did not have enough time to ask all the questions they wanted. In these situations, e-consultations were found to be useful for sending questions after the consultation.

## Discussion

Our findings emphasise the need for a sufficient level of digital health literacy for older patients to be able to learn about and use e-consultations. It has been shown that older patients may have the motivation to use digital health care services but lack skills and trust in new technologies and need help and guidance to be able to access them [28,29]. Our study elaborates on the picture by showing how the respondents, who had a fair level of digital health literacy feared losing their digital literacy over time, thus also losing their ability to use digital health care services.

The respondents, who were older patients with high health care needs, found e-consultations useful to seek health care and receive clarifications about their health problems, confirming the findings from other studies [3,8,28,30,31]. The results emphasise that patients must voluntarily decide when and for what e-consultations should be used to perceive the service as good.

The availability of e-consultations was highly appreciated by the respondents, who often felt that the GP and the GP office were unavailable due to long waiting times on the phone. Satisfaction with the availability of e-consultations has also been shown in earlier studies [1,11]. In the current study, e-consultations were seen as a new possibility to contact the GP as well as a way for the patients to get help assessing whether to book a physical appointment. Similar mechanisms have been reported in England [32] and Denmark [11], where e-consultations were perceived as a direct line to the GP, fast-tracking past the gate-keeping function of the front desk and not used as a replacement for physical consultations [32]. The patients in our study had expectations of receiving a response from the GP within one to two days, thus confirming the expectations shown in earlier studies [2,11,28].

Finally, our study showed that e-consultations were perceived as safe if patients trusted their GP through an established relationship. Studies from other countries have also emphasised the relationship between patients and GP when using e-consultations [12,30].

### How is the access to e-consultations for older patients?

The findings on the use of e-consultations by older patients were further interpreted through Levesque’s theoretical perspective on access. There are several points to be made about the accessibility of the service. First, because not all GPs in Norway offer the service, there are patients who do not have the opportunity to send e-consultations to their GP. This makes the service unequally accessible for patients, depending on the offer from their GP. Secondly, because this study explored the experiences of users of the service, access had already been achieved for the respondents. However, when looking at the findings through the lens of Levesque’s framework of access, we identified important areas of potential improvement of the service that can contribute to offering a more equally accessible service to all older patients.

#### Ability to perceive (approachability)

The patients’ ability to perceive that the service existed relied on the patients becoming aware of e-consultations by exploring the digital portal, which required a certain level of digital health literacy. No outreaching information about the opportunity to use e-consultation was given to patients by the GP or the GP office. Poor information about the benefits of digital health care services and lack of functional assistance are documented barriers for older patients [9,33].

#### Ability to seek (acceptability)

The patients’ ability to seek access to health care through e-consultations relies on the patients feeling safe by doing so, and not facing conflicts with social or cultural beliefs. Digital health literacy helps the patients’ ability to seek, as a high level of digital health literacy contributes to creating trust in digital spheres. In addition, our study showed that for the e-consultations to be accepted, the use should be voluntary and self-chosen, and unlimited access to physical consultation forms must also be offered. This confirmed results from other studies, which showed that digital health care services are more easily accepted when they are considered a complement and not a substitute for physical consultations [34,35]. Our study also found that some patients felt a duty to book an e-consultation rather than a physical consultation to reduce the GP’s workload. This could affect the acceptability of the service.

#### Ability to reach (availability)

The patients’ ability to reach their GP through e-consultations at any time of the day was perceived as an important factor for using the service. The service was seen as a new way to contact the GP, as well as help to assess whether it was necessary to book an appointment at the GP office. The high availability of e-consultations created a lower threshold of reaching for help.

#### Ability to pay (affordability)

No concerns were raised regarding the patients’ ability to pay for e-consultations or other digital devices, nor about the cost of an e-consultation being the same as a physical consultation. Patients with high health care needs often receive an exemption card relatively early in the year, which was also the case for many respondents in this study. This might have influenced their perception of the affordability of the service.

#### Ability to engage (appropriateness)

The patients’ ability to engage depended on their wish for self-managing their health issues. Our study showed that e-consultations could lead to increased empowerment by giving patients the opportunity to access health care when and if they want easy clarifications. E-consultations were not used for critical issues, as the format was not deemed appropriate for such purposes by the patients. Patients who had a trusting relationship with their GP perceived the outcome of e-consultations as appropriate and safe.

### Strengths and weaknesses

The respondents of this study had a relatively high level of digital health literacy compared to the general population of older patients. This might have affected the results and their transferability to older patients in general. However, we believe that the results seen in context of access theory give a valuable understanding of the service's accessibility. This knowledge can also be used to improve access to the service among older patients with a lower level of digital health literacy. Despite a very diverse recruitment strategy, it was hard to find men with experience in using e-consultations who wanted to be interviewed. The results might therefore be influenced by the larger proportion of women among the respondents.

The interviews were conducted by phone, with the awareness that phone interviews have disadvantages compared to interviews in person (e.g. lack of a personal atmosphere and observation of body language). However, the awareness of creating a safe environment was present in the interview setting, and the interviews were introduced with a long, open sequence about the respondents’ lives. This open start gave the respondents a good warm-up and created a safe atmosphere. Moreover, the phone contact eliminated unnecessary travel and the risk of COVID-19, whilst facilitating the opportunity to include participants from across the whole country.

The diverse background and expertise of the interdisciplinary research team has supported the investigator triangulation in the data interpretation process and strengthened the quality of the study.

### Possible mechanisms and implications for clinicians or policymakers

E-consultations should be accessible for all patients, and our study raises some concerns about the equity of access. Promotion of the service to patients and clarity on its suitability are currently missing in Norway. Previous studies have showen that the main reason for not using a digital health care service is not being aware of the service [9,33]. Our study showed that the way patients learn about e-consultations relies on their digital health literacy and ability to find out about the service. Information about the service should be available and shared with all patients to avoid dependence on individual competence, thus helping older patients to access and use digital services [9,29,33].

The GP scheme in Norway consider physical consultations as the main form of consultation, thus ensuring that non-digital patients have access to health care through physical services. E-consultations are not suitable for all health issues or for all patients, and e-consultations could therefore never fully replace physical consultations as the only or main access to health care [1]. We need to be conscious of how capacity problems and high workload burdens for GPs affect patient’s assessment of the choice of consultation form. Available recommendations on how to use e-consultations could help patients choose the proper consultation form and, at the same time, enable their self-involvement and assessment of suitability [14]. Recommendations must state that all issues can be discussed at the GP office in person to prevent the perception that some issues are only accepted through e-consultations [14].

Our study exposed that, in addition to clinical inquiries, many patients used e-consultations to get help assessing whether a problem should be investigated through a physical appointment, or it could wait. Triaging health care problems to the appropriate level of care is traditionally done by a health care secretary at the front desk of the GP office or by phone. By using e-consultations for triaging health care problems, patients solve the problem of long waiting times on the phone. At the same time, this way of using the service may result in an additional workload for the GP, as it shifts work from the health secretaries to the GP.

Users expected a short response time on e-consultations (i.e. within two days), whilst the current national legislation states that an inquiry should normally be answered within five days [22]. If the overall workload for GPs increases, this will most likely lead to a longer response time for e-consultations. A longer response time could, in turn, lead to a less satisfying service for the patients and lower patient safety if potentially acute issues are not handled within a brief period of time. The maximum response time and the unsuitability of use for acute issues must be communicated clearly. Assigning designated time slots of the GP’s time to handle e-consultations [35] or limiting the number of e-consultations that each GP can receive in one day, may be a solution to maintain a short response time and manage demand [36].

## Supplementary Material

Supplemental Material

## Footnotes

1 Only combined statistics for video consultations, telephone consultations and text-based e-consultations are available.

2 The average number of consultations with a GP per year per patient is three in Norway.
