# Supplementary material for: Older patients’ experiences of access to and use of e-consultations with the general practitioner in Norway: an interview study
Source: Scand J Prim Health Care. 2023 Jan 2;41(1):33–42. doi: 10.1080/02813432.2022.2161307 (PMC10088919; doi:10.1080/02813432.2022.2161307)
Supplement: Supplemental Material [file IPRI_A_2161307_SM5894.docx]

# Supplemental online material

Supplement 1. Interview guide.

*Introduction*

“I would like to start by getting to know you and your everyday life a little”

- Are you a pensioner - how long have you been a pensioner? (age)

- When did you last work and what did you work as? / What do you work with?

- How is your everyday life (as a pensioner)?

*Health needs*

- How is your need for health care?

- Can you tell me about the last time you were (physically) at the GP?

- How often do you contact your GP?

- How do you think your doctor’s availability is?

*Digital health literacy*

- How do you like to use digital technology?

- How did you find out that there was an opportunity to use e-consultations to contact you GP?

*Use of e-consultations*

- Can you tell me about the last time you wrote a text consultation to your GP?

- Why did you choose to do a e-consultation instead of other consultation form?

- What do you consider when choosing whether to send a e-consultation, or directly book an hour for a physical meeting?

- How did you perceive the doctor’s understanding of the problem/issues you described in the e-consultation?

- What do you think about the e-consultations in comparison to a regular physical consultation in regard to communication?

- In what contexts do you consider it useful to send the doctor a e-consultation instead of meeting at the office?

- Who do you think should decide whether a consultation should be digital or physical?

- Do you think the doctor’s availability has changed after you started using text? If yes, how?

- Would you describe an e-consultation is a supplement or a substitute for a traditional consultation?

- How do you word yourself in a text consultation?

- How do you experience the communication between you and the doctor after the first message?

*Overall – sum up*

- What do you think is positive about doing e-consultations?

- What do you find difficult / challenging about e-consultations?

- In general, what do you think about health care being provided over the internet (video / text) and not in the same room as the health personnel?

*The future*

- How do you think you will use digital solutions at your GP in the future?

- That is all from me, are there anything you would want to add?
